# Associations among Wine Grape Microbiome, Metabolome, and Fermentation Behavior Suggest Microbial Contribution to Regional Wine Characteristics

**DOI:** 10.1128/mBio.00631-16

**Published:** 2016-06-14

**Authors:** Nicholas A. Bokulich, Thomas S. Collins, Chad Masarweh, Greg Allen, Hildegarde Heymann, Susan E. Ebeler, David A. Mills

**Affiliations:** a Department of Food Science and Technology, University of California, Davis, California, USA; b Department of Viticulture and Enology, University of California, Davis, California, USA; c Foods for Health Institute, University of California, Davis, California, USA; d Food Safety and Measurement Facility, University of California, Davis, California, USA; e Far Niente and Nickel & Nickel Wineries, Oakville, California, USA

## Abstract

Regionally distinct wine characteristics (*terroir*) are an important aspect of wine production and consumer appreciation. Microbial activity is an integral part of wine production, and grape and wine microbiota present regionally defined patterns associated with vineyard and climatic conditions, but the degree to which these microbial patterns associate with the chemical composition of wine is unclear. Through a longitudinal survey of over 200 commercial wine fermentations, we demonstrate that both grape microbiota and wine metabolite profiles distinguish viticultural area designations and individual vineyards within Napa and Sonoma Counties, California. Associations among wine microbiota and fermentation characteristics suggest new links between microbiota, fermentation performance, and wine properties. The bacterial and fungal consortia of wine fermentations, composed from vineyard and winery sources, correlate with the chemical composition of the finished wines and predict metabolite abundances in finished wines using machine learning models. The use of postharvest microbiota as an early predictor of wine chemical composition is unprecedented and potentially poses a new paradigm for quality control of agricultural products. These findings add further evidence that microbial activity is associated with wine *terroir*.

## IMPORTANCE

Wine production is a multi-billion-dollar global industry for which microbial control and wine chemical composition are crucial aspects of quality. *Terroir* is an important feature of consumer appreciation and wine culture, but the many factors that contribute to *terroir* are nebulous. We show that grape and wine microbiota exhibit regional patterns that correlate with wine chemical composition, suggesting that the grape microbiome may influence *terroir*. In addition to enriching our understanding of how growing region and wine properties interact, this may provide further economic incentive for agricultural and enological practices that maintain regional microbial biodiversity.

## INTRODUCTION

Regional variations in wine quality traits, collectively referred to as “*terroir*,” have been documented empirically for centuries, but few conclusive links have been drawn between regional factors and wine sensory properties. Wines made from identical grape cultivars but grown in different regions are appreciated for their distinctive features, increasing the consumer demand for and economic value of many regional products. Consequently, geographical pedigree is legally protected, e.g., through the Protected Designation of Origin regulations in Europe and American Viticultural Areas (AVAs) in the United States. Moreover, such regionality can be distinguished by chemical composition (1 – 3) and sensory characteristics (4 – 7), but establishing which conditions drive quality outcomes remains tenuous. These chemico-sensory differences are most commonly ascribed to environmental factors that influence grapevine growth and development, involving interactions between environmental, temporal, geologic, plant-genetic, human, and other factors (8). Thus, *terroir* is an important aspect of consumer acceptance, identity, and economic appreciation of wine production. Defining factors that contribute to *terroir* is important for preserving the diversity and enhancing the value of wine and other regional agricultural commodities.

Microbial biogeography is another factor that potentially contributes to regional wine characteristics. Traditional winemaking practices encourage or rely entirely on “native” (noninoculated) microbiota to conduct fermentations, a practice that adherents regard as enhancing regional typicity. In spite of the well-defined role microbial interactions play in grapevine health, fruit quality, and wine quality (9), the influence of grape microbiota on regional characteristics of wines is undefined. We previously demonstrated that regional, grape varietal, and climatic factors shape the bacterial and fungal communities of wine grapes across multiple growing years (10). Other authors have demonstrated regional fungal biodiversity patterns on grapes elsewhere globally (11 – 13). Regional strains of *Saccharomyces cerevisiae*, the principal yeast species involved in wine fermentations, produce distinct wine chemical compositions, demonstrating one prominent route by which regional microbes influence *terroir* (14). Beyond *Saccharomyces* yeasts, wine fermentation is a complex, multispecies process, and the synergistic effects of these consortia on wine chemistry are yet unclear. An overwhelming body of evidence has defined the influences of numerous bacteria and fungi on the chemical and sensory properties of wines in pure culture (reviewed in reference 15), and nonfermentative grape-associated microbiota produce many sensory-active compounds associated with wine aroma, highlighting their potential in early flavor formation (16). However, the relationship between regional microbial patterns and wine metabolite profiles is unknown. Evidence of their interaction would implicate microbial activity in shaping the regional wine qualities that are important for defining product identity.

Furthermore, high-throughput sequencing techniques have expanded our knowledge of microbial diversity on grapes and in wine fermentations, but the possible roles and dynamics of these microbes during wine fermentation are understudied (10, 13, 17 – 19). In addition to directly influencing wine chemical composition, understudied microbes could indirectly alter wine quality—e.g., by inhibiting fermentation progress or malolactic fermentations.

**FIG 1:**
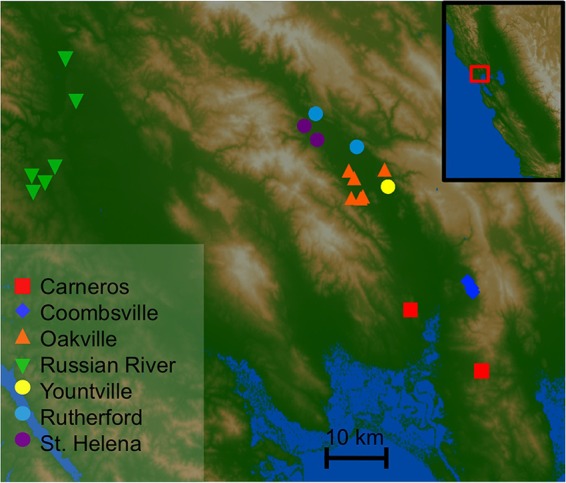
Map of sampling sites across Napa and Sonoma Counties. Each point represents an individual vineyard from which grapes were harvested for the fermentations monitored in this study. Points are colored by AVA designation, as indicated in the key. The inset illustrates the position of this sampling area within California.

To address these issues, we conducted an exploratory study to assess (i) whether the grape microbiota and wine metabolomes exhibit distinct patterns of distribution at small geographic scales (e.g., neighboring vineyards), (ii) whether regional wine microbiomes and metabolomes are correlated, and (iii) associations between the microbiome, fermentation performance, and prefermentation grape must/juice characteristics. We employed high-throughput marker gene sequencing to longitudinally profile the bacterial and fungal consortia of over 200 commercial fermentations and musts (crushed grapes) of grapes grown throughout Napa and Sonoma Counties, CA (Fig. 1; see Table S1 in the supplemental material). We used ultra-high-pressure liquid chromatography (UHPLC)/quadrupole time of flight mass spectrometry (QTOF MS) for nontargeted metabolite profiling of a subset of these must and wine samples, identifying marker metabolites that differentiate AVAs. We demonstrate that the grape/wine microbiota and metabolites are regionally distinct, the must and wine microbiota correlate with the wine metabolome and fermentation performance, and grape must microbial composition predicts the metabolite composition of the finished wine, suggesting that microbial dispersion patterns may contribute to regional wine characteristics.

## RESULTS AND DISCUSSION

All samples were collected from Far Niente and Nickel & Nickel wineries, located approximately 2 km apart in Oakville, CA (Napa County). Cabernet Sauvignon (dry red wine) and Chardonnay (dry white wine) grape musts and fermentations were longitudinally sampled across fermentation and aging (Table 1). Red and white wine fermentations were sampled at different time points, as they are processed differently: white grapes are crushed and pressed immediately, and the clarified juices are fermented, whereas red grapes are crushed and fermented as must, which is only pressed after fermentation is complete (Table 1). Additionally, only the red wines underwent malolactic fermentation (MLF), a secondary bacterial fermentation during which *Oenococcus oeni* and other lactic acid bacteria deacidify wine by conversion of malic to lactic acid, accompanied by various sensory changes.

**TABLE 1:** Fermentation stages and sample collection schematic

Stage^a^	Process	Chardonnay		Cabernet Sauvignon	
		Status^b^	Site^c^	Status	Site
Crush	Crush	Juice	PP		
Must	Prior to inoculation	Juice	T	Must	T
Early	Early fermentation	~22 °Brix	T		
Mid	Mid-fermentation			~20 °Brix	T
Late	Near end of fermentation	~1 °Brix	B	Pressed	T
End	End of fermentation pre-SO_2_	Dry (wine)	B		
MLF	End of MLF			ML—dry	B
Wine	Aged	Prior to bottling	B	Prior to racking	B

a Stage name indicates the name used in subsequent figures.

b Status indicates the material type and sugar concentration (°Brix) at that stage. Musts are unfermented crushed grapes containing both pomace and juice. Juice is unfermented, pressed grape must. The product is considered wine after the end of fermentation (“end” stage). Empty entries indicate no sample collected at that stage, as red and white wines are processed differently.

c PP, press pan; T, fermentation tank; B, barrel; MLF, malolactic fermentation.

### Microbial biodiversity distinguishes vineyards and viticultural areas (AVAs).

We have previously demonstrated that different grape-growing regions of California possess distinct, identifiable microbial patterns across large distances, correlated with local weather conditions (10). Thus, we first sought to test whether microbial patterns can be distinguished between contiguous AVAs and individual vineyards within a single growing region, Napa County, CA, and nearby sites in Sonoma County at different stages of fermentation (Fig. 1; see Table S1 in the supplemental material).

**FIG 2:**
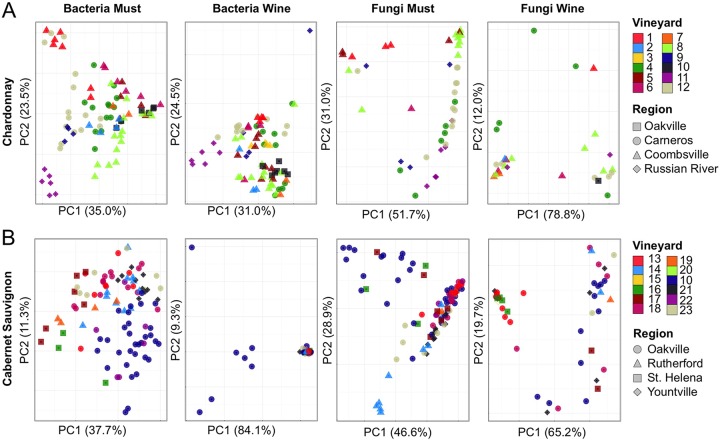
Microbiota exhibits regional variation in musts and wines. (A) Chardonnay. (B) Cabernet Sauvignon. Shown are PCoA comparisons of bacterial weighted UniFrac distance (left two columns) and fungal Bray-Curtis dissimilarity (right two columns) in musts and wines (see column labels), categorized by vineyard (color) and AVA source (shape). Each point represents an individual sample, and sample proximity on the plot is a function of similarity in bacterial and fungal community composition.

Individual AVAs and vineyards were distinguished based on the microbial consortia present in the grape must/juice (Fig. 2; see Table S2 in the supplemental material). Permutational multivariate analysis of variance (MANOVA) tests (see Table S2) confirmed that microbial composition is significantly different between at least two AVAs (Chardonnay bacteria, *P* < 0.001, *R*^2^ = 0.262, and fungi, *P* < 0.001, *R*^2^ = 0.233; Cabernet bacteria, *P* < 0.001, *R^2^* = 0.154, and fungi, *P* = 0.002, *R*^2^ = 0.105) and vineyards (Chardonnay bacteria, *P* < 0.001, *R*^2^ = 0.599, and fungi, *P* < 0.001, *R*^2^ = 0.408; Cabernet bacteria, *P* < 0.001, *R*^2^ = 0.353, and fungi, *P* < 0.001, *R*^2^ = 0.320). Random forest machine learning models confirm that all vineyards are distinguishable at classification accuracies between 79% (Chardonnay juice) and 82% (Chardonnay wine), 3.7- to 4.4-fold more accurate than random error rates (see Table S3 in the supplemental material). This separation was also dependent upon the grape variety: Chardonnay demonstrated stronger AVA differentiation for both bacterial and fungal profiles than Cabernet Sauvignon (Fig. 2; see Table S2). Thus, local conditions appear to modulate microbial communities in addition to regional effects. Numerous microclimatic, viticultural, and geophysical factors could explain variation among vineyard sites beyond the scope of our measurements and are important questions for future studies. Intravineyard monitoring could elucidate which of these factors hold the greatest influence over localized microbial patterns, potentially yielding insight into manipulable elements for controlling local microbial communities: e.g., to reduce disease pressure or increase plant-beneficial populations.

**FIG 3:**
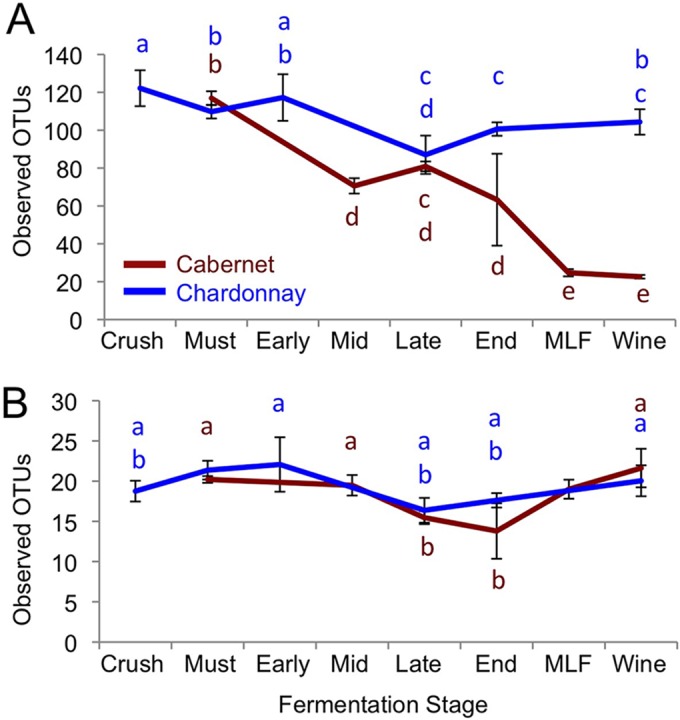
Stage of fermentation influences microbial richness. Shown are the mean ± standard deviation (SD) bacterial (A) and fungal (B) richness (observed OTU) in Cabernet Sauvignon and Chardonnay by stage of fermentation. Different lowercase letters indicate significantly different means.

**FIG 4:**
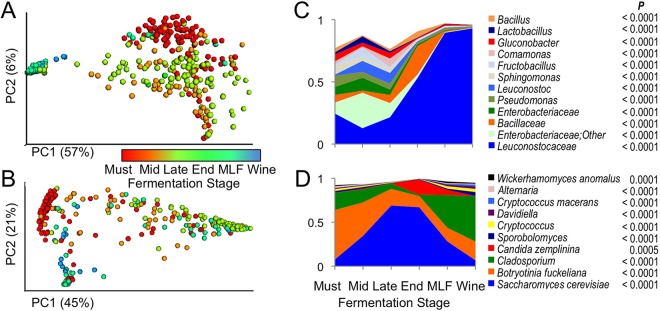
Stage of fermentation influences microbial composition of Cabernet Sauvignon. (A and B) Bacterial weighted UniFrac (A) and fungal Bray-Curtis (B) PCoA comparing similarity among all Cabernet samples, colored by stage of fermentation. (C and D) Relative abundance of bacteria (C) and fungi (D) that differed significantly by stage of fermentation. Only taxa detected at >1% relative abundance are shown in panels C and D. False discovery rate (FDR)-corrected *P* values are listed for each taxon*.* The taxon “*Leuconostocaceae*” represents *O. oeni*, which belongs to this bacterial family, and all OTU assigned to this taxonomy matched *O. oeni* by BLASTn.

Both AVA and vineyard-specific microbial signatures diminished during fermentation (Fig. 2) as growth of fermentative organisms reshaped the community structure, richness, and diversity of the wines (Fig. 3 and Fig. 4; see Fig. S1 in the supplemental material). This effect was largely dependent on grape variety and winery: Chardonnay vineyards and AVAs retained significantly different bacterial profiles at end of fermentation (*P* < 0.001) (Fig. 2; see Table S2 and Fig. S1 in the supplemental material) and Cabernet fungi differentiated vineyard origin of at least one vineyard (*P* < 0.001) (Fig. 2 and 4; see Table S2), but Cabernet bacterial profiles became less distinct due to growth of *Leuconostocaceae* (*O. oeni*) during MLF conducted in these wines but not in the Chardonnays. Nevertheless, random forest classification models could still distinguish vineyards at accuracies of 81% (Cabernet) and 82% (Chardonnay) based on microbial profiles in the finished wine, indicating that vineyard-specific signatures are still retained through fermentation (see Table S3 in the supplemental material).

### Wine metabolite profiles segregate growing regions.

We next sought to test whether AVAs and vineyards produced differentiable wine metabolite patterns, and whether regional microbial patterns could translate to metabolomic differences in wines. Using ultra-high-pressure liquid chromatography (UHPLC)/quadrupole time of flight mass spectrometry (QTOF MS), we analyzed the metabolite profiles of 13 Chardonnay and 27 Cabernet Sauvignon wines in triplicate, representing distinct AVAs and vineyards tested with biological replication (minimum duplicate). These were finished and barreled but unblended fermentations (MLF stage for Cabernets, and “end” stage for Chardonnays [Table 1]), enabling metabolite profiles to be compared directly to the microbial communities inhabiting the musts from which these wines were made. All vineyards and AVAs were represented by biological replicates: i.e., at least two separate vineyard blocks were analyzed per vineyard, and at least two separate vineyards were analyzed per AVA whenever possible.

Raw QTOF profiles revealed 1,585 mass features in Cabernet Sauvignon wines and 1,054 in Chardonnay wines. Profiles were filtered to remove putative metabolites that were not observed consistently across technical replicates or detected in low abundance. Only low-molecular-mass putative metabolites (<300 *m/z*) were analyzed to focus on compounds that are most likely aroma-active volatile compounds. (Larger compounds were primarily identified as grape-derived phenolic compounds and ignored in this study.) Of the remaining putative metabolites, we retained only those observed at significantly different abundances between regions (one-way analysis of variance [ANOVA] false discovery rate [FDR]-corrected *P* value of <0.05). In all, Cabernet Sauvignon wines contained 16 regionally differential low-mass features (see Table S4 in the supplemental material). Chardonnay wines contained 27 (see Table S5 in the supplemental material). In several cases, exact identities could be confidently determined by mass and tandem MS (MS/MS) spectrum matches to the metabolite databases or accurate run time and mass matches to authentic standards. In most cases, only approximate identities or no identity could be obtained. This is a common issue, as the QTOF analysis as used here is a nontargeted method and the reference databases are not tailored to wine metabolites. Many of these compounds represent acids, esters, and aldehydes, some of which are likely microbial. Others, such as tartaric acid, are strictly grape derived. Many of the grape-derived compounds, such as the phenolic compounds coumaric acid, gallic acid, catechin, epicatechin, and caffeic acid, are modified by microbial metabolism during wine fermentation (20 – 22).

Within grape varieties, wine metabolite profiles clearly splay out with principal-component analysis (PCA), associated with numerous significantly discriminant metabolites (FDR-corrected *P* value of <0.05) (see Fig. S2 in the supplemental material). Chardonnay wines demonstrated greater discrimination between both growing regions and vineyards (see Fig. S2A), whereas separation was weaker among Cabernet Sauvignon wines, for which many vineyards were indistinguishable from each other (see Fig. S2B). The reasons why Chardonnay microbiota better differentiate region are unclear, but the use of MLF in all Cabernet wines studied (but not the Chardonnays) is one possibility. The Chardonnay vineyards sampled in this study also came from more distant and diverse regions across Napa and Sonoma Counties (e.g., Carneros, Coombsville, Oakville, Russian River), compared to Cabernet (St. Helena, Oakville, Rutherford, and Yountville are contiguous regions on the valley floor), and thus differences in climate, topography, and regional distance are all possible causes that cannot be unraveled in the present study.

### Microbial patterns correlate to regional metabolite profiles.

**FIG 5:**
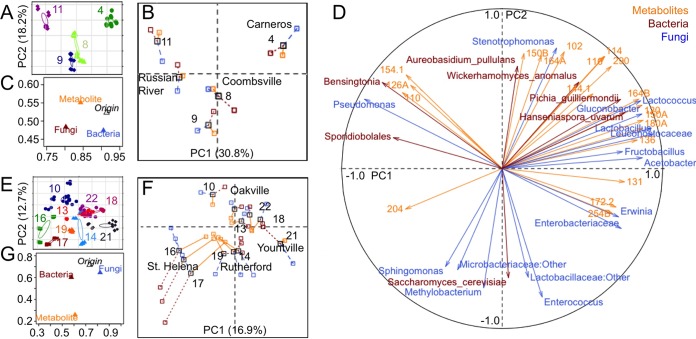
Must microbiome is correlated with the wine metabolome. Shown are the results from multifactorial analysis (MFA) of must microbiome and wine metabolome profiles of Chardonnay (A to D) and Cabernet Sauvignon (E to G). (A and E) An MFA sample ordination plot demonstrates regional and vineyard segregation of Chardonnay (A) and Cabernet Sauvignon (E). (B and F) A partial-axis MFA plot illustrates category correspondence between metabolite (orange), bacterial (blue), and fungal (red) subcategory coordinates. Partial mean individuals (means of sample ordination for bacterial, fungal, or metabolite profiles) are linked to the subcategory common mean (centroid of all samples for a given region of origin). (C and G) MFA group representations illustrate the relationship between bacterial, fungal, and metabolite profiles with wine origin (region and vineyard). (D) An MFA correlation circle depicts correlations in normalized abundance between all Chardonnay must bacterial taxa (blue), fungal taxa (red), and wine metabolites (orange) along the MFA axes. Metabolite nominal masses are used for clarity; accurate masses are provided in Table S5 in the supplemental material. To improve readability of the plots, only the top correlations in each dimension are shown.

To further dissect the relationship between regionally differential must microbiota and wine metabolites, multifactorial analysis (MFA) (23) was used to investigate underlying relationships between putative metabolite profiles, grape microbiota, and region of origin (Fig. 5). MFA is a generalization of principal-component analysis (PCA), in which sample similarity is decided by multiple different sets of observations (in this case, both metabolites and taxonomic features) to determine a consensus ordination. This analysis calculates sample similarity (as ordination plots akin to PCA), the degree of similarity between each set of observations, and correlations between individual observations. MFA could confidently separate wines by growing region and vineyard based on putative metabolite profiles and grape microbiota (Fig. 5A and E). Highly similar ordination patterns of regional and site-specific segregation were observed based on metabolite, bacterial, and fungal profiles (Fig. 5B and F), and numerous correlations were detected between variables in all three groups (Fig. 5C and G), demonstrating close correspondence between microbial and metabolic profiles. In Chardonnay wines, fungal profiles were more closely associated with putative metabolite profiles than with region alone (Fig. 5C), and several interesting correlations emerged between the microbiome and metabolome (Fig. 5D): notably, between *Leuconostocaceae* (*O. oeni* is the top BLAST hit for all *Leuconostocaceae* sequences detected) and entity 136.0498@0.9307 (i.e., accurate mass of 136.0498 at LC run time of 0.9307 min) (possible hits, methylbenzoate, phenyl acetate, or *p*-anisealdehyde); *Hanseniaspora uvarum* and entity 120.0577@3.3848 (possible hits, acetophenone, phenylacetaldehyde, or 3-methyl benzaldehyde), and *Pichia guilliermondii* and entities 144.1169@7.3606 (octanoic acid) and 114.0702@2.2747 (C_6_H_10_O_2_ acid, ester, or lactone) (Fig. 5D). These microbes are all known fermentative organisms (some with poorly characterized phenotypes); these putative metabolites are all important sensory-active wine components or potentially sensory-active metabolites, and all are correlated by MFA with vineyards in Carneros, one of the most renowned, cold-weather AVAs for Chardonnay production within Napa County.

Cabernet Sauvignon bacterial profiles were more closely associated with putative metabolite profiles than with region alone (Fig. 5G), and there was a weaker correlation between fungal and metabolic profiles (Fig. 5F). The stronger bacterium-metabolite correlation may reflect that the Cabernet Sauvignons underwent MLF (a bacterial fermentation) and longer maturation, muting fungal contributions. Hence, wine production methods and wine style may ultimately determine the degree to which different microbial activities contribute to wine chemical composition. The close correspondence between must microbial composition and putative wine metabolite profiles may indicate that the grape microbiota influences the chemical properties of the finished wine and/or that both are strongly shaped by the same regional factors.

### Microbial profiles predict abundance of wine metabolites.

**FIG 6:**
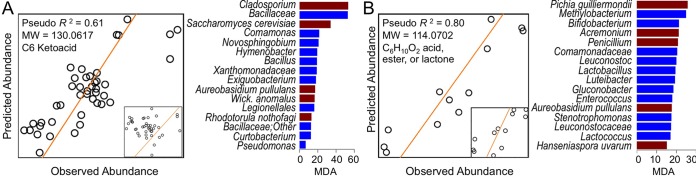
Microbial composition accurately predicts metabolite abundance of select metabolites. Shown are the results from random forest regression of predicted versus observed metabolite intensity in validation samples using a sparse set of predictors (inset, model using all taxa as predictors) to predict abundance of a C_6_ keto acid in Cabernet Sauvignon wines (A) and a C_6_ acid, ester, or lactone in Chardonnay wines (B). Trend lines indicate a true 1:1 ratio. Bar plots were used to display the ranked feature importance of bacterial (blue bars) and fungal (red bars) taxa used to train the optimized models. MW, molecular weight; MDA, mean decrease in model accuracy if that feature is removed from the model.

Wine is a complex chemical and biological matrix, and many of the sensory-active constituents are produced, consumed, or modified by multiple microbial species (15). Relationships between microbial composition and wine metabolite profiles are unlikely to be one dimensional and linear. Thus, models incorporating multiple microbial predictors can be expected to more accurately predict such relationships. We employed random forest (24) classification models to predict wine metabolite composition as a function of grape must microbiome composition. Within the metabolome, a few select putative wine metabolites were predicted relatively well by these models (pseudo-*R^2^* = 0.50 to 0.98) (Fig. 6; see Tables S6 and S7 in the supplemental material). Metabolite entity 130.0617@3.1301 (pseudo-*R^2^* = 0.61), a C_6_ ketoacid, is best predicted by the presence of the grape-associated filamentous fungus *Cladosporium* and *Bacillaceae*; fermentative yeasts *S. cerevisiae* and *Wickerhamomyces anomalus* were also top features in the optimized predictive model (Fig. 6A)*. Pichia guilliermondii*, closely correlated by MFA with entity 114.0702@2.2747 (C_6_H_10_O_2_ acid, ester, or lactone), is the principal feature for predicting abundance of this metabolite in Chardonnays (pseudo-*R^2^* = 0.80) (Fig. 6B). Several other regionally discriminant metabolites were closely linked to microbiota composition (see Tables S6  and S7). For many of these metabolites, the top predictive features include fermentative yeasts and bacteria such as *Saccharomyces*, *H. uvarum*, *W. anomalus*, *P. guilliermondii*, *Lactobacillales*, and *Acetobacteraceae*, and dominant grape epiphytes, such as *Cladosporium*, *Botryotinia fuckeliana*, *Bacillaceae*, *Enterobacteriaceae*, *Pseudomonas*, *Sphingomonas*, and *Methylobacterium* (see Tables S6  and S7). Metabolites predicted with high accuracy include mass features 136.0498@0.9307 (pseudo-*R^2^* = 0.83; possible hits, methylbenzoate, phenyl acetate, or *p*-anisealdehyde), 120.0577@3.3848 (pseudo-*R^2^* = 0.86; possible hits, acetophenone, phenylacetaldehyde, or 3-methyl benzaldehyde), 204.1179@5.5837 (pseudo-*R^2^* = 0.85; possible mercaptol hexyl butyrates), 272.1797@5.7748 (pseudo-*R^2^* = 0.89; unknown compound), and 164.0446@1.4888 (pseudo-*R^2^* = 0.97; coumaric acid) (see Tables S6 and 7). The important sensory-active medium-chain fatty acid octanoic acid was predicted moderately well in Chardonnay (entity 144.1169@7.3606; pseudo-*R^2^* = 0.53).

Many of these microbiota-metabolite associations corroborate the well-defined metabolic characteristics of these organisms in pure culture fermentations (15). For the many organisms with unknown roles in wine fermentations, these results raise suggestive associations between grape microbiota and wine chemistry. The grape epiphytes in particular are not well studied for their potential contributions to wine, although they are numerically dominant on grapes and in early fermentations. We recently cultured two of these bacteria, *Sphingomonas* and *Methylobacterium*, from finished wines (17), and *Enterobacteriaceae*, *Pseudomonas*, *Sphingomonas*, and *Methylobacterium* appear to increase in relative abundance during fermentation (not necessarily an indication of growth) in this study and the work of others (13, 18, 19, 25), increasing the probability that these bacteria contribute to wine characteristics directly or indirectly. Plant-associated, nonfermentative, and numerically minor populations could still exert a substantial effect on metabolite profiles directly—e.g., through prefermentation activity or release of metabolites with low sensory thresholds—and indirectly through metabolism or release of small molecules and enzymes following cell death and lysis. Interactions among regional microbiota may also influence the metabolome and deserve further study. For example, release of inhibitory molecules by less fermentative organisms present in grape musts could alter *Saccharomyces* metabolism, altering wine profiles. However, the DNA profiling techniques as employed here do not differentiate active populations within the microbiota, yielding little insight on such relationships. Metatranscriptomics would distinguish the functional behavior of active populations during the fermentations, inferring whether populations make direct versus indirect contributions to wine metabolite profiles and bolstering the microbiota-metabolite associations detected here.

While these findings cannot claim causation, they demonstrate that the microbial composition of grapes accurately predicts the chemical composition of wines made from these grapes and are therefore biomarkers for predicting wine metabolite composition (a quantifiable feature of *terroir*). An alternative explanation for the correlation between the prefermentation microbiome and wine metabolome is that both are influenced by the same regional factors (e.g., climate or soil chemistry) and by the metabolite profiles of the grapes. More work must be done to establish the possible roles of these organisms in wine fermentation and flavor production, and sensory studies are necessary to determine whether microbial associations extend to human-perceptible differences in wine traits. We provide a rich exploratory data set that will support mechanistic studies focusing on the roles of these understudied organisms in wine fermentations.

### Microbial profiles correlate with juice chemistry and fermentation behavior.

Next, we sought to detect correlations between microbiota, must/juice chemistry, and fermentation characteristics, with an emphasis on detecting microbiota that could influence fermentation behavior. Correlations between the microbiota, fermentation rate, and MLF length are particularly important for discovering factors that may contribute to sluggish or stuck fermentations (26).

**FIG 7:**
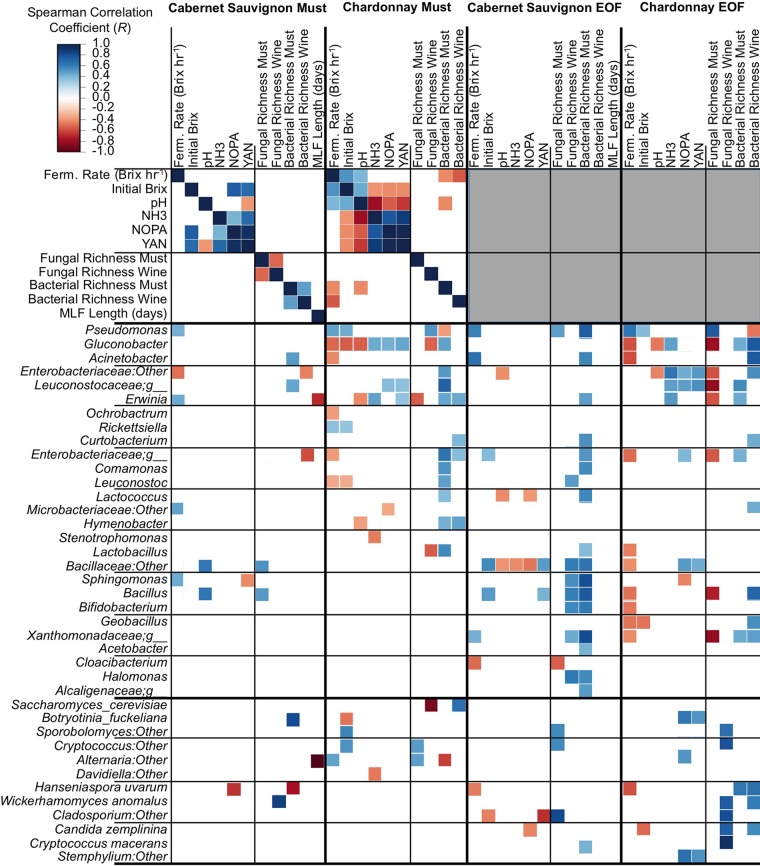
Microbiota correlations with must/juice and fermentation characteristics. Shown is the Spearman correlation between must/juice chemistry, fermentation characteristics, and microbiota in musts (left columns) and end of fermentations (EOF [right columns]). Only significant correlations (FDR-corrected *P* value of <0.05) are shown. As chemical composition was only measured in musts and juices, no data appear for must/juice correlations at end of fermentation (gray boxes at top right corner). NH3, ammonia concentration; NOPA, total nitrogen by *o*-phthaldialdehyde assay; YAN, yeast assimilable nitrogen.

Numerous significant correlations (FDR-corrected Spearman *P* value of <0.05) were detected between initial must/juice composition and microbial abundance in musts and at the end of fermentation (Fig. 7). Notably, fermentation rate was negatively correlated with several taxa, as well as bacterial richness in Chardonnay musts and wines, suggesting that high bacterial diversity inhibits alcoholic fermentation, most likely because greater richness increases the chances of antagonistic species being present. Among the negatively correlated taxa, *H. uvarum*, *Gluconobacter*, and *Lactobacillus* spp. are already known to inhibit fermentation rate through competition for nutrients with *S. cerevisiae* (26, 27), so recovery of these correlations is reassuring. The repeated negative correlation between *Enterobacteriaceae* and fermentation rate in musts and wines is particularly suggestive of another potential interaction, although the species involved will need to be clarified. (The group detected here represents operational taxonomic units [OTU] identifiable only to the family level.) *Erwinia* and other *Enterobacteriaceae* have been observed abundantly in botrytized (17, 25) and table wine fermentations (13, 18, 19), but the nature and role of this group in wines have been unclear. Various *Enterobacteriaceae* are typically present in and contribute to the flavor of some spontaneous beer fermentations (28) but hinder the fermentation rate in beer (29) and could be similarly problematic in some wine fermentations. Several other organisms were positively correlated with fermentation rate, most notably *Pseudomonas*, which was significantly correlated in both Cabernet and Chardonnay, both in musts and at the end of fermentation (Fig. 7). The possibility that this bacterium may enhance fermentation rate is an interesting finding worth further investigation.

### Conclusions.

Microbial *terroir* likely involves multiple interacting aspects of microbial distribution, strain diversity, and plant-microbial interactions. The present study explores issues of regional distribution of microbial populations in grapes and wines, building on previous evidence that these patterns exist over larger regions, correlating with climate conditions (10). Not all regions and vineyards are microbiologically unique, and the patterns that distinguish them are not random. Instead, climate and distance between regions are associated with regional microbial patterns (10), and many other factors are likely involved, including processes that are selective (e.g., soil type, topography, human-driven agricultural practices) and neutral (e.g., species dispersal limitation across large distances) (30). Vineyard soil microbiota demonstrate similar regional distribution patterns, associated instead with edaphic factors (31, 32), and plant-microbial interactions above and below ground may contribute to plant growth and development, leading to changes in fruit quality (32). Geographic distribution of microbial strains displaying diverse phenotypes appears to be another factor (14). Knight and coworkers (14) found that *S. cerevisiae* genotypes and phenotypes were correlated with geographic dispersion in New Zealand, and regional strains produced distinct metabolite profiles in experimental wine fermentations. Regional strain diversity may also explain dispersion of wine spoilage traits, such as geographic patterns of histamine decarboxylase genes in lactic acid bacteria in wineries across Bordeaux, France (33). Regional strain diversity in the many other bacterial and fungal species involved in wine production may similarly contribute to microbial *terroir* and deserves further investigation.

The intricacies of wine flavor are not determined by microbial composition alone. We conjecture that microbial activity contributes to the mixture of abiotic and biotic factors that underlie wine *terroir*, with the scale of this contribution depending upon the winemaking techniques and style of wine produced. We have demonstrated that the microbial constituents of grape musts are biomarkers for predicting features of wine metabolite composition before fermentation has even commenced. These markers could provide actionable information to winemakers to improve wine characteristics or mitigate problem fermentations—and are unprecedented as early predictors of the wine metabolome. Such information could be practical for predicting the suitability of potential vineyard sites or acquisitions or for preventing microbiological issues in abnormal vintages. We doubt that microbial biomarkers could be used to artificially replicate all aspects of wine *terroir*, as many other interacting, nonmanipulable factors also contribute (e.g., climate). *Terroir* is the regional fingerprint of a wine, not solely an engineered feature of winemaking.

Wine is a useful model for testing the theory of microbial *terroir*, as regional wine qualities are already a well-recognized and celebrated part of wine identity. However, the connection between microbial regionality and food properties is not unique to wine, and these results suggest that similar phenomena likely occur in other food products. Thus, these findings argue for exploring and characterizing the connection between environmental conditions, microbial patterns, and chemico-sensory characteristics in other agricultural products (both food and nonfood) impacted by microbial activities. Microbial *terroir* may provide further incentive for preserving regional biodiversity through sustainable agricultural practices, in recognition of the economic values provided by regional product identity (14).

Together, these results illustrate a complex relationship between microbial communities in grape musts and wine fermentations with the chemical compositions of the resulting wines. Microbial communities can be distinguished on regional, AVA, and vineyard-specific scales, correlating with multiple environmental parameters (10). The microbial consortium of wine fermentation, influenced by vineyard and winery sources (34), is associated with the chemical composition of the finished wine, suggesting that—if indeed the microbial connection is causative and these changes result in sensory-active effects—microbial biogeography is a quantitative, definable feature of wine *terroir*. We identify numerous associations between the wine microbiome and metabolome, and future studies are necessary to establish causative links between the microbial consortia, wine metabolites, and sensory characteristics.

## MATERIALS AND METHODS

### Sampling and DNA extraction.

Samples were collected from Far Niente Winery and Nickel & Nickel Winery, both located in Oakville, Napa County, CA. All samples were collected from the 2011 vintage. These wineries use grapes harvested from throughout Napa County, representing several major viticultural areas (Fig. 1; see Table S1 in the supplemental material). The primary wine and grape varieties collected were Chardonnay (a dry white wine) and Cabernet Sauvignon (a dry red wine).

Samples consisted of longitudinal wine fermentation samples (*n* = 777), each corresponding to individual vineyard lots. Samples were collected at five predetermined time points in duplicate. As red and white grapes are processed differently, these times points depended on grape type (Table 1). Red grape fermentations were collected as grape must (destemmed, crushed grapes prior to fermentation), at mid-fermentation, at the end of fermentation following pressing but prior to barreling, at the end of malolactic fermentation (in barrels), and after several months of barrel aging. White grape fermentations were collected as juice, following racking (clarification) prior to inoculation, early fermentation prior to barreling, near the end of fermentation (in barrels), at the end of fermentation (in barrels), and after several months of maturation in barrels.

Samples were frozen immediately, shipped on ice, and stored at −80°C until processing. Sample processing was performed as described previously (17). Briefly, must samples were thawed and centrifuged at 4,000 × *g* for 15 min, washed 3 times in ice-cold phosphate-buffered saline (PBS), suspended in 200 µl DNeasy lysis buffer (20 mM Tris-Cl [pH 8.0], 2 mM sodium EDTA, 1.2% Triton X-100) supplemented with 40 mg/ml lysozyme, and incubated at 37°C for 30 min. From this point, the extraction proceeded following the protocol of the Qiagen fecal DNA extraction kit protocol (Qiagen, Valencia, CA), with the addition of a bead beater cell lysis step of 2 min at maximum speed using a FastPrep-24 bead beater (MP Bio, Solon, OH). DNA extracts were stored at −20°C until further analysis.

### Sequencing library construction.

Amplification and sequencing were performed as described previously for analysis of bacterial (10) and fungal (35) communities. Briefly, the V4 domain of bacterial 16S rRNA genes was amplified using primers F515 (5′-NNNNNNNN**GT**GTGCCAGCMGCCGCGGTAA-3′) and R806 (5′-GGACTACHVGGGTWTCTAAT-3′) (36), with the forward primer modified to contain a unique 8-nucleotide (nt) bar code (italicized poly-N section of primer above) and 2-nt linker sequence (boldface portion) at the 5′ terminus. PCR mixtures contained 5 to 100 ng DNA template, 1× GoTaq Green master mix (Promega), 1 mM MgCl_2_, and 2 pmol of each primer. Reaction conditions consisted of an initial 94°C for 3 min, followed by 35 cycles of 94°C for 45 s, 50°C for 60 s, and 72°C for 90 s, and a final extension of 72°C for 10 min. Fungal internal transcribed spacer 1 (ITS1) loci were amplified with primers BITS (5′-NNNNNNNN**CT**ACCTGCGGARGGATCA-3′) and B58S3 (5′-GAGATCCRTTGYTRAAAGTT-3′) (35), with a unique 8-nt bar code and linker sequence incorporated in each forward primer. PCR mixtures contained 5 to 100 ng DNA template, 1× GoTaq Green master mix (Promega, Madison, WI), 1 mM MgCl_2_, and 2 pmol of each primer. Reaction conditions consisted of an initial 95°C for 2 min, followed by 40 cycles of 95°C for 30 s, 55°C for 30 s, and 72°C for 60 s, and a final extension of 72°C for 5 min. Amplicons were combined into two separate pooled samples (keeping bacterial and fungal amplicons separate) at roughly equal amplification intensity ratios, purified using the Qiaquick spin kit (Qiagen), and submitted to the UC, Davis Genome Center DNA Technologies Core for Illumina paired-end library preparation, cluster generation, and 250-bp paired-end sequencing on an Illumina MiSeq instrument in two separate runs.

### Data analysis.

Raw Illumina fastq files were demultiplexed, quality filtered, and analyzed using QIIME v1.7.0 (37). Reads were truncated at any site containing >3 consecutive bases receiving a quality score of <1e−5, and any read containing one or more ambiguous base calls was discarded, as were truncated reads of <190 nt. Operational taxonomic units (OTU) were assigned using QIIME’s uclust-based (38) open-reference OTU-picking work flow, with a threshold of 97% pairwise identity. Sequence prefiltering (discarding sequences with <60% pairwise identity to any reference sequence) and reference-based OTU picking were performed using a representative subset of the greengenes bacterial 16S rRNA database (13_5 release) (39) or the UNITE fungal internal transcribed spacer (ITS) database (9_12 release) (40), filtered to remove incomplete and unannotated taxonomies (35). OTU were classified taxonomically using the RDP classifier (41). Bacterial 16S rRNA gene sequences were aligned using PyNAST (42) against a template alignment of the greengenes core set filtered at 97% similarity. From this alignment, chimeric sequences were identified and removed using ChimeraSlayer (43), and a phylogenic tree was generated from the filtered alignment using FastTree (44). Sequences failing alignment or identified as chimeric were removed prior to downstream analysis. Any OTU representing less than 0.001% of the total filtered sequences was removed to avoid inclusion of erroneous reads, leading to inflated estimates of diversity (45), as were samples represented by less than 500 (bacterial) or 100 (fungal) sequences following all quality-filtering steps.

Beta-diversity (similarity between samples) was calculated within QIIME using the weighted UniFrac (46) distance between samples (evenly sampled at 1,000 reads per sample) to assess similarity among bacterial communities and Bray-Curtis dissimilarity for fungal communities. Principal coordinates were computed from the resulting distance matrices to compress dimensionality intro three-dimensional principal-coordinate analysis (PCoA) plots, enabling visualization of sample relationships. In order to determine whether sample classifications (AVA, variety, and vineyard) contained differences in phylogenetic diversity, permutational MANOVA (47) with 999 permutations was used to test significant differences between sample groups based on weighted UniFrac (bacterial) or Bray-Curtis (fungal) distance matrices. For all categorical classifications (AVA, variety, and vineyard) rejecting this null hypothesis, Kruskal-Wallis tests were used to determine which taxa differed between sample groups.

All other statistical tests were performed in R software (v 2.15.0). Principal-component analysis (PCA) and multifactorial analysis (MFA) (23) were performed in R with the FactoMineR package (48) to assess regional variations between wine metabolites, must and wine microbiota, and regional affiliations. Only metabolites and taxa demonstrating significant regional differences (ANOVA and Kruskal-Wallis FDR-corrected *P* value of <0.05, respectively) were used in MFA and random forest analyses.

Random forest (24) supervised learning models were employed to predict wine metabolite composition as a function of must microbial composition (regression model), to predict region of origin as a function of microbial composition (classification model), and to predict region of origin as a function of metabolite composition (classification). Model predictions were made using the out-of-bag error cross-validation, whereby random samples are removed from the model one by one (with replacement) and used to cross-validate the prediction accuracy of the restrained model. Models were optimized using 100-fold cross-validation to select the minimal number of features (taxa or metabolites) necessary to minimize prediction error, and secondary models were trained using only these features. The resulting feature importance for each feature (metabolite or taxon) describes the relative importance of that feature to that model, quantified as the increase in mean square error when that feature is removed from the prediction model.

### UHPLC/QTOF MS.

A set of 13 Chardonnay and 27 Cabernet Sauvignon wines were selected for metabolite profiling, representing the main growing AVAs and vineyards analyzed in this study in biological replicates. Samples were diluted 1:4 in molecular-grade deionized water analyzed in triplicate in random order. A control was analyzed in quadruplicate at the start of the sample set, in triplicate at the end of the sample set, and singly after every tenth sample to monitor for variation in mass accuracy and retention time. Each sample was analyzed in an untargeted data-dependent MS/MS mode after the individual sample’s triplicate single MS analyses were done, as described below.

Chromatography was performed using an Agilent 1290 ultra-high-pressure liquid chromatograph (UHPLC) coupled to an Agilent 6530 quadrupole time of flight mass spectrometer QTOF MS) (Agilent Technologies, Santa Clara, CA). Triplicate 5-µl injections were tested for each sample. Chromatographic separation was accomplished using a Zorbax Eclipse Plus C_18_ column (5-cm by 2.1-mm inside diameter [i.d.], 1.8-µm particle size; Agilent Technologies, Santa Clara, CA). The column heater was set to 60°C throughout the analysis. A reversed-phase gradient was used, with 0.1% acetic acid in water as mobile phase A and 20% mobile phase A–80% HPLC-grade methanol as mobile phase B. From an initial condition of 97% A–3% B, the concentrations changed in a linear gradient from 97% A at 1.00 min to 100% B at 9.00 min, followed by a second linear gradient change from 100% B at 10.00 min to 97% A at 11.00 min, with a total time of 12.00 min for each analysis. The mobile phase flow rate was held at 0.6 ml/min throughout the analysis. An Agilent Jet Stream dual-spray electrospray ionization (ESI) source was used in negative mode to focus the liquid chromatography (LC) effluent and separately introduce reference compounds to generate ions for analysis. The system was calibrated using the manufacturer’s recommended procedure prior to each analysis run. Mass spectral data were acquired in profile and centroid mode over an *m*/*z* range from 100 to 1,700; the transient accumulation rate was 1.41 spectra/s. The ESI source parameters were as follows: drying gas temperature, 350°C; drying gas flow rate, 10 liters/min; nebulizer pressure, 45 lb/in^2^; sheath gas temperature, 400°C; sheath gas flow rate, 11 liters/min; capillary voltage, 3,000 V; and nozzle voltage, 1,000 V. The QTOF MS settings were fragmentor voltage, 175 V, skimmer voltage, 65 V, and octopole 1 radio frequency (RF) voltage, 750 V. A reference solution containing purine and hexakis(1*H*,1*H*,3*H*-tetrafluoropropoxy)phosphazine (HP-921) was continuously introduced just prior to the ESI source using an isocratic pump into a separate sprayer of the dual Jet Stream source, producing signals of *m*/*z* 119.0362 (proton-abstracted purine) and 980.0164 (acetate adduct of HP-921) in negative mode; these signals were used for continuous internal mass calibration throughout the analysis, in order to ensure high mass accuracy for ions detected during the analysis.

MS/MS profiling was conducted in the automated data-dependent MS/MS mode, using a collision energy of 20 eV for all compounds. The Jet Stream ESI source settings were the same as for the QTOF MS experiments. The MS data were collected with an acquisition rate of 3 spectra/s over a mass range of 75 to 1,500 *m*/*z*. A maximum of two precursor ions were allowed for each MS scan. The minimum precursor threshold of 200 counts was used, and precursors were selected based on their abundance. Active exclusion was enabled to exclude precursor ions after 2 spectra were collected, so that spectra of other suitable precursor ions could also be collected. Excluded precursor ions were released after 0.1 min, so that spectra of isomers with differing retention times could be collected if present. The MS/MS data were collected with an acquisition rate of 3.0 spectra/s over an MS/MS mass range of 50 to 1,450 *m*/*z*, with a medium isolation width of ~4 Da: the isolation width was 0.3 Da to the left of the precursor ion and 3.7 Da to the right, which allowed for the inclusion of the isotopes of the precursor ion in the collision cell.

Raw LC-MS data were processed using the Agilent MassHunter Qualitative Analysis software, version 6.00 (Agilent Technologies, Inc., Santa Clara, CA) to mine the data for the presence of nonredundant mass features using isotope peaks, for the presence of adduct ions, to eliminate noise, and to filter those entities found with a minimum abundance (peaks with counts of ≥1 × 10^6^). Raw MS profiles were processed using the Agilent Mass Profiler Professional (MPP) software, version 12.6 (Agilent Technologies, Inc.), to align mass and retention time data across the samples within the set and to define profiling parameters. Only low-mass entities (≤300 *m*/*z*) were retained for analysis, in order to focus on mass features that are most likely microbial and are most likely aroma active. Mass features displaying inconsistent presence/absence of detection across replicates for any sample were removed, and all remaining mass features were tested using one-way ANOVA with FDR correction, retaining only mass features that differed significantly between AVAs (FDR-corrected *P* value of <0.05). PCA and MFA of mass feature data were performed in R, as described above. Putative compound identities were obtained based on matches to the METLIN metabolite database (https://metlin.scripps.edu). A 30-ppm mass window was used for identification, and putative identifications were based on matching the accurate mass, the retention time, and MS/MS spectra of a given entity to those available in databases. When possible, identifications were confirmed with authentic standards.

### Data availability.

All raw marker gene sequencing data have been publicly deposited in the QIITA database (http://qiita.ucsd.edu) under accession no. 10119 (http://qiita.ucsd.edu/study/description/10119) and in EBI under accession number ERP015814.

## SUPPLEMENTAL MATERIAL

FIGURE S1  Fermentation stage exhibits winery-specific influences on Chardonnay microbial profiles. (A and B) Bacterial weighted UniFrac PCoA of Far Niente Chardonnay fermentations, color coded by stage (A), and Nickel & Nickel Chardonnay color coded and labeled by vineyard of origin (B). (C) Fungal Bray-Curtis dissimilarity of all Chardonnay samples colored by stage indicates that fungal profiles change by stage of fermentation and follow the same pattern in both wineries. (D and E) Relative abundance of bacteria (D) and fungi (E) that differed significantly by stage of fermentation. Only taxa detected at >1% relative abundance are shown. False discovery rate (FDR)-corrected *P* values are listed for each taxon*.* C, crush stage; M, must; E, early fermentation. Download Figure S1, JPG file, 0.5 MB

FIGURE S2  Metabolite profiles of Chardonnay and Cabernet Sauvignon wines cluster by vineyard and AVA. PCA of metabolite profiles of Chardonnay (A) and Cabernet Sauvignon (B) wines categorized by vineyard (color) and AVA source (shape). Download Figure S2, JPG file, 0.3 MB

TABLE S1  Number of must and juice samples collected from each vineyard. Table S1, DOCX file, 0.01 MB

TABLE S2  Permutational MANOVA comparisons of microbial diversity in musts and wines. Table S2, DOCX file, 0.01 MB

TABLE S3  Random forest models predict vineyard origin of grape musts. Table S3, DOCX file, 0.05 MB

TABLE S4  Regionally differential Cabernet Sauvignon wine mass features and putative metabolites. Table S4, DOCX file, 0.01 MB

TABLE S5  Regionally differential chardonnay wine mass features and putative metabolites. Table S5, DOCX file, 0.01 MB

TABLE S6  Chardonnay metabolite random forest model summaries. Table S6, DOCX file, 0.01 MB

TABLE S7  Cabernet Sauvignon metabolite random forest model summaries. Table S7, DOCX file, 0.01 MB

## References

[1] Pereira GE, Gaudillere JP, van Leeuwen C, Hilbert G, Lavialle O, Maucourt M, Deborde C, Moing A, Rolin D 2005 ^1^H NMR and chemometrics to characterize mature grape berries in four wine-growing areas in Bordeaux, France. J Agric Food Chem 53:6382–6389. doi:10.1021/jf058058q. 16076122

[2] Son HS, Hwang GS, Kim KM, Ahn HJ, Park WM, van den Berg F, Hong YS, Lee CH 2009 Metabolic studies on geographical grapes and their wines using ^1^H NMR analysis coupled with multivariate statistics. J Agric Food Chem 57:1481–1490. doi:10.1021/jf803388w. 19192969

[3] López-Rituerto E, Savorani F, Avenoza A, Busto JH, Peregrina JM, Engelsen SB 2012 Investigations of La Rioja terroir for wine production using ^1^H NMR metabolomics. J Agric Food Chem 60:3452–3461. doi:10.1021/jf204361d. 22397579

[4] Fischer U, Roth D, Christmann M 1999 The impact of geographic origin, vintage, and wine estate on sensory properties of *Vitis vinifera* cv. Riesling wines. Food Qual Preference 10:281–288. doi:10.1016/S0950-3293(99)00008-7.

[5] Robinson AL, Adams DO, Boss PK, Heymann H, Solomon PS, Trengove RD 2012 Influence of geographic origin on the sensory characteristics and wine composition of *Vitis vinifera* cv. Cabernet sauvignon wines from Australia. Am J Enol Vitic 63:467–476. doi:10.5344/ajev.2012.12023.

[6] Heymann H, Noble A 1987 Descriptive analysis of commercial Cabernet sauvignon wines from California. Am J Enol Vitic 38:41–44.

[7] Kontkanen D, Reynolds AG, Cliff MA, King M 2005 Canadian terroir: sensory characterization of Bordeaux-style red wine varieties in the Niagara Peninsula. Food Res Int 38:417–425. doi:10.1016/j.foodres.2004.10.010.

[8] Van Leeuwen C, Seguin G 2006 The concept of terroir in viticulture. J Wine Res 17:1–10. doi:10.1080/09571260600633135.

[9] Barata A, Malfeito-Ferreira M, Loureiro V 2012 The microbial ecology of wine grape berries. Int J Food Microbiol 153:243–259. doi:10.1016/j.ijfoodmicro.2011.11.025. 22189021

[10] Bokulich NA, Thorngate JH, Richardson PM, Mills DA 2014 Microbial biogeography of wine grapes is conditioned by cultivar, vintage, and climate. Proc Natl Acad Sci U S A 111:E139–E148. doi:10.1073/pnas.1317377110. 24277822 PMC3890796

[11] Taylor MW, Tsai P, Anfang N, Ross HA, Goddard MR 2014 Pyrosequencing reveals regional differences in fruit-associated fungal communities. Environ Microbiol 16:2848–2858. doi:10.1111/1462-2920.12456. 24650123 PMC4257574

[12] Gayevskiy V, Goddard MR 2012 Geographic delineations of yeast communities and populations associated with vines and wines in New Zealand. ISME J 6:1281–1290. doi:10.1038/ismej.2011.195. 22189497 PMC3379632

[13] Pinto C, Pinho D, Cardoso R, Custódio V, Fernandes J, Sousa S, Pinheiro M, Egas C, Gomes AC 2015 Wine fermentation microbiome: a landscape from different Portuguese wine appellations. Front Microbiol 6:905. doi:10.3389/fmicb.2015.00905. 26388852 PMC4555975

[14] Knight S, Klaere S, Fedrizzi B, Goddard MR 2015 Regional microbial signatures positively correlate with differential wine phenotypes: evidence for a microbial aspect to terroir. Sci Rep 5:14233. doi:10.1038/srep14233. 26400688 PMC4585847

[15] Swiegers JH, Bartowsky EJ, Henschke PA, Pretorius IS 2005 Yeast and bacterial modulation of wine aroma and flavour. Aust J Grape Wine Res 11:139–173. doi:10.1111/j.1755-0238.2005.tb00285.x.

[16] Verginer M, Leitner E, Berg G 2010 Production of volatile metabolites by grape-associated microorganisms. J Agric Food Chem 58:8344–8350. doi:10.1021/jf100393w. 20575540

[17] Bokulich NA, Joseph CM, Allen G, Benson AK, Mills DA 2012 Next-generation sequencing reveals significant bacterial diversity of botrytized wine. PLoS One 7:e36357. doi:10.1371/journal.pone.0036357. 22563494 PMC3341366

[18] Piao H, Hawley E, Kopf S, DeScenzo R, Sealock S, Henick-Kling T, Hess M 2015 Insights into the bacterial community and its temporal succession during the fermentation of wine grapes. Front Microbiol 6:809. doi:10.3389/fmicb.2015.00809. 26347718 PMC4539513

[19] Bokulich NA, Swadener M, Sakamoto K, Mills DA, Bisson LF 2015 Sulfur dioxide treatment alters wine microbial diversity and fermentation progression in a dose-dependent fashion. Am J Enol Viticulture 66:73–79. doi:10.5344/ajev.2014.14096.

[20] Chatonnet P, Dubourdie D, Boidron J, Pons M 1992 The origin of ethylphenols in wines. J Sci Food Agric 60:165–178. doi:10.1002/jsfa.2740600205.

[21] Alberto MR, Gómez-Cordovés C, Manca de Nadra MC 2004 Metabolism of gallic acid and catechin by *Lactobacillus hilgardii* from wine. J Agric Food Chem 52:6465–6469. doi:10.1021/jf049239f. 15479008

[22] Rodríguez H, Curiel JA, Landete JM, de las Rivas B, López de Felipe F, Gómez-Cordovés C, Mancheño JM, Muñoz R 2009 Food phenolics and lactic acid bacteria. Int J Food Microbiol 132:79–90. doi:10.1016/j.ijfoodmicro.2009.03.025. 19419788

[23] Escofier B, Pages J 1990 Multiple factor analysis. Comput Stat Data Anal 18:121–140.

[24] Breiman L 2001 Random Forests. Mach Learn 45:5–32. doi:10.1023/A:1010933404324.

[25] Nisiotou AA, Rantsiou K, Iliopoulos V, Cocolin L, Nychas GJ 2011 Bacterial species associated with sound and *Botrytis*-infected grapes from a Greek vineyard. Int J Food Microbiol 145:432–436. doi:10.1016/j.ijfoodmicro.2011.01.017. 21315469

[26] Bisson LF 1999 Stuck and sluggish fermentations. Am J Enol Vitic 50:107–119.

[27] Fleet GH 2003 Yeast interactions and wine flavour. Int J Food Microbiol 86:11–22. doi:10.1016/s0168-1605(03)00245-9. 12892919

[28] Bokulich NA, Bamforth CW 2013 The microbiology of malting and brewing. Microbiol Mol Biol Rev 77:157–172. doi:10.1128/MMBR.00060-12. 23699253 PMC3668669

[29] Priest FG, Cowbourne MA, Hough JS 1974 Wort enterobacteria—review. J Inst Brew 80:342–356. doi:10.1002/j.2050-0416.1974.tb03629.x.

[30] Morrison-Whittle P, Goddard MR 2015 Quantifying the relative roles of selective and neutral processes in defining eukaryotic microbial communities. ISME J 9:2003–2011. doi:10.1038/ismej.2015.18. 25756681 PMC4542032

[31] Burns KN, Kluepfel DA, Strauss SL, Bokulich NA, Cantu D, Steenwerth KL 2015 Vineyard soil bacterial diversity and composition revealed by 16S rRNA genes: differentiation by geographic features. Soil Biol Biochem 91:232–247. doi:10.1038/ismej.2015.18.

[32] Zarraonaindia I, Owens SM, Weisenhorn P, West K, Hampton-Marcell J, Lax S, Bokulich NA, Mills DA, Martin G, Taghavi S, van der Lelie D, Gilbert JA 2015 The soil microbiome influences grapevine-associated microbiota. mBio 6:e02527-14. doi:10.1128/nBio.02527-14. 25805735 PMC4453523

[33] Lucas PM, Claisse O, Lonvaud-Funel A 2008 High frequency of histamine-producing bacteria in the enological environment and instability of the histidine decarboxylase production phenotype. Appl Environ Microbiol 74:811–817. doi:10.1128/aem.01496-07. 18065614 PMC2227711

[34] Bokulich NA, Ohta M, Richardson PM, Mills DA 2013 Monitoring seasonal changes in winery-resident microbiota. PLoS One 8:e66437. doi:10.1371/journal.pone.0066437. 23840468 PMC3686677

[35] Bokulich NA, Mills DA 2013 Improved selection of internal transcribed spacer-specific primers enables quantitative, ultra-high-throughput profiling of fungal communities. Appl Environ Microbiol 79:2519–2526. doi:10.1128/AEM.03870-12. 23377949 PMC3623200

[36] Caporaso JG, Lauber CL, Walters WA, Berg-Lyons D, Lozupone CA, Turnbaugh PJ, Fierer N, Knight R 2011 Global patterns of 16S rRNA diversity at a depth of millions of sequences per sample. Proc Natl Acad Sci U S A 108:4516–4522. doi:10.1073/pnas.1000080107. 20534432 PMC3063599

[37] Caporaso JG, Kuczynski J, Stombaugh J, Bittinger K, Bushman FD, Costello EK, Fierer N, Gonzalez Pena AG, Goodrich JK, Gordon JI, Huttley GA, Kelley ST, Knights D, Koenig JE, Ley RE, Lozupone CA, McDonald D, Muegge BD, Pirrung M, Reeder J, Sevinsky JR, Turnbaugh PJ, Walters WA, Widmann J, Yatsunenko T, Zaneveld J, Knight R 2010 Qiime allows analysis of high-throughput community sequence data. Nat Methods 7:335–336. doi:10.1038/nmeth.f.303. 20383131 PMC3156573

[38] Edgar RC 2010 Search and clustering orders of magnitude faster than BLAST. Bioinformatics 26:2460–2461. doi:10.1093/bioinformatics/btq461. 20709691

[39] DeSantis TZ, Hugenholtz P, Larsen N, Rojas M, Brodie EL, Keller K, Huber T, Dalevi D, Hu P, Andersen GL 2006 Greengenes, a chimera-checked 16S rRNA gene database and workbench compatible with ARB. Appl Environ Microbiol 72:5069–5072. doi:10.1128/AEM.03006-05. 16820507 PMC1489311

[40] Abarenkov K, Henrik Nilsson R, Larsson K, Alexander IJ, Eberhardt U, Erland S, Høiland K, Kjøller R, Larsson E, Pennanen T, Sen R, Taylor AFS, Tedersoo L, Ursing BM, Vrålstad T, Liimatainen K, Peintner U, Kõljalg U 2010 The UNITE database for molecular identification of fungi—recent updates and future perspectives. New Phytol 186:281–285. doi:10.1111/j.1469-8137.2009.03160.x. 20409185

[41] Wang Q, Garrity GM, Tiedje JM, Cole JR 2007 Naive Bayesian classifier for rapid assignment of rRNA sequences into the new bacterial taxonomy. Appl Environ Microbiol 73:5261–5267. doi:10.1128/AEM.00062-07. 17586664 PMC1950982

[42] Caporaso JG, Bittinger K, Bushman FD, DeSantis TZ, Andersen GL, Knight R 2010 PyNAST: a flexible tool for aligning sequences to a template alignment. Bioinformatics 26:266–267. doi:10.1093/bioinformatics/btp636. 19914921 PMC2804299

[43] Haas BJ, Gevers D, Earl AM, Feldgarden M, Ward DV, Giannoukos G, Ciulla D, Tabbaa D, Highlander SK, Sodergren E, Methé B, DeSantis TZ, Petrosino JF, Knight R, Birren BW 2011 Chimeric 16S rRNA sequence formation and detection in Sanger and 454-pyrosequenced PCR amplicons. Genome Res 21:494–504. doi:10.1101/gr.112730.110. 21212162 PMC3044863

[44] Price MN, Dehal PS, Arkin AP 2010 FastTree 2—approximately maximum-likelihood trees for large alignments. PLoS One 5:e9490. doi:10.1371/journal.pone.0009490. 20224823 PMC2835736

[45] Bokulich NA, Subramanian S, Faith JJ, Gevers D, Gordon JI, Knight R, Mills DA, Caporaso JG 2013 Quality-filtering vastly improves diversity estimates from Illumina amplicon sequencing. Nat Methods 10:57–59. doi:10.1038/nmeth.2276. 23202435 PMC3531572

[46] Lozupone C, Knight R 2005 UniFrac: a new phylogenetic method for comparing microbial communities. Appl Environ Microbiol 71:8228–8235. doi:10.1128/AEM.71.12.8228-8235.2005. 16332807 PMC1317376

[47] Anderson MJ 2001 A new method for non-parametric multivariate analysis of variance. Aust Ecol 26:32–46. doi:10.1046/j.1442-9993.2001.01070.x.

[48] Le S, Josse J, Husson F 2008 FactoMineR: an R package for multivariate analysis. J Stat Soft 25:1–18.

