# Supplementary figures and images for: Associations among Wine Grape Microbiome, Metabolome, and Fermentation Behavior Suggest Microbial Contribution to Regional Wine Characteristics
Source: mBio. 2016 Jun 14;7(3):e00631-16. doi: 10.1128/mBio.00631-16 (PMC4959672; doi:10.1128/mBio.00631-16)

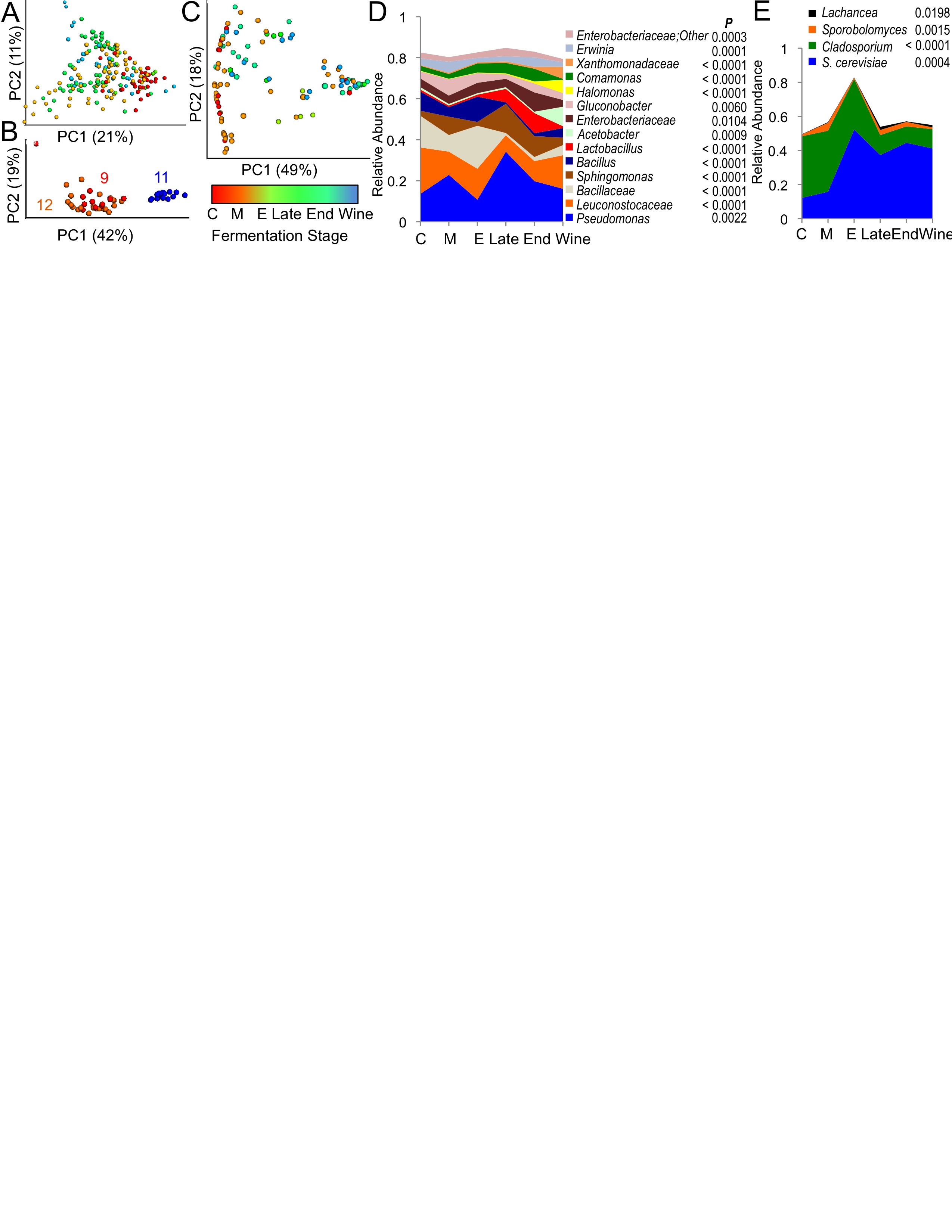

Supplement: FIGURE S1 — Fermentation stage exhibits winery-specific influences on Chardonnay microbial profiles. (A and B) Bacterial weighted UniFrac PCoA of Far Niente Chardonnay fermentations, color coded by stage (A), and Nickel & Nickel Chardonnay color coded and labeled by vineyard of origin (B). (C) Fungal Bray-Curtis dissimilarity of all Chardonnay samples colored by stage indicates that fungal profiles change by stage of fermentation and follow the same pattern in both wineries. (D and E) Relative abundance of bacteria (D) and fungi (E) that differed significantly by stage of fermentation. Only taxa detected at >1% relative abundance are shown. False discovery rate (FDR)-corrected P values are listed for each taxon. C, crush stage; M, must; E, early fermentation. Download [file mbo003162841sf1.jpg]

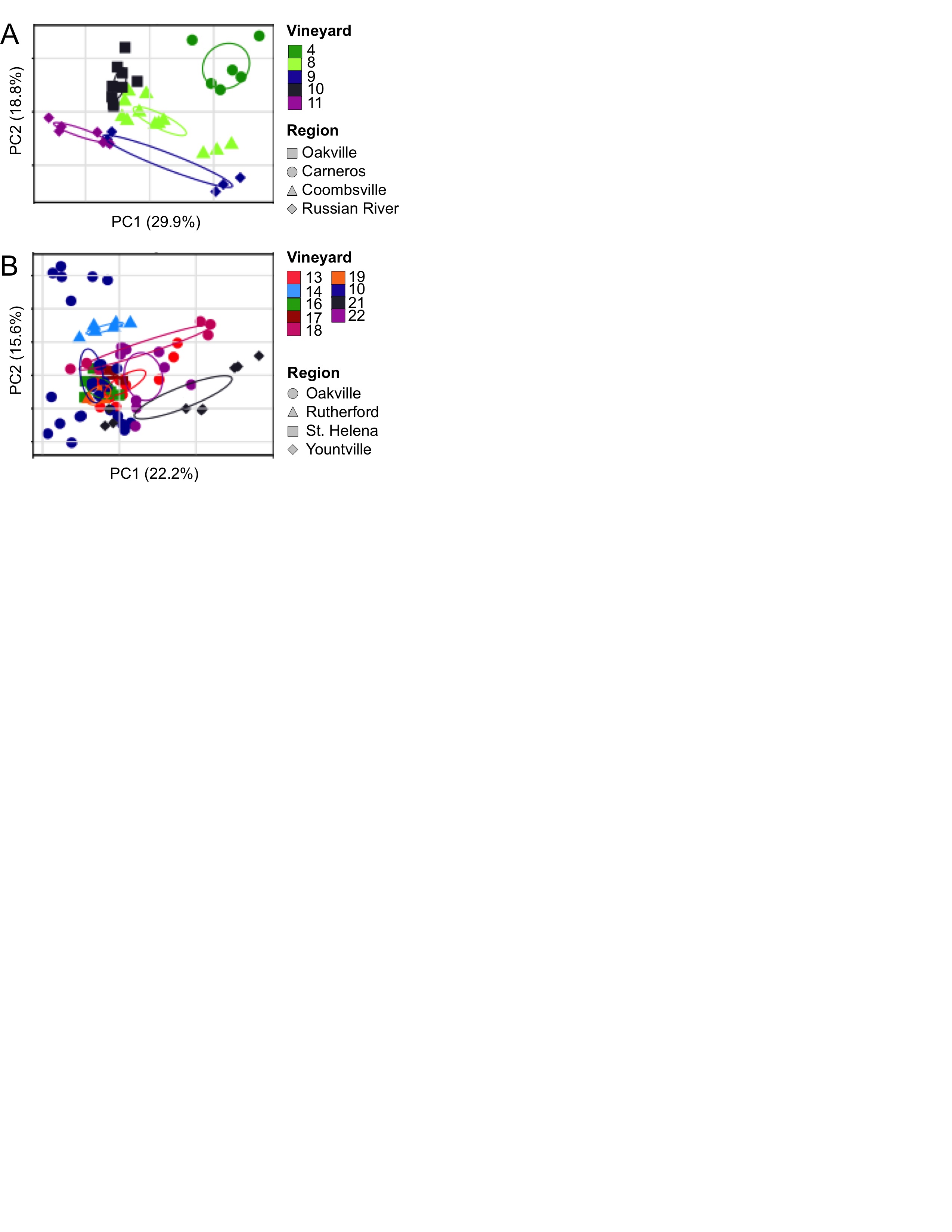

Supplement: FIGURE S2 — Metabolite profiles of Chardonnay and Cabernet Sauvignon wines cluster by vineyard and AVA. PCA of metabolite profiles of Chardonnay (A) and Cabernet Sauvignon (B) wines categorized by vineyard (color) and AVA source (shape). Download [file mbo003162841sf2.jpg]
