# Supplementary material for: Associations among Wine Grape Microbiome, Metabolome, and Fermentation Behavior Suggest Microbial Contribution to Regional Wine Characteristics
Source: mBio. 2016 Jun 14;7(3):e00631-16. doi: 10.1128/mBio.00631-16 (PMC4959672; doi:10.1128/mBio.00631-16)
Supplement: TABLE S1 — Number of must and juice samples collected from each vineyard. [file mbo003162841st1.docx]

**Table S1. Number of Must and Juice Samples Collected from Each Vineyard**

| Winery* | Variety | Region | Vineyard | Count |
| --- | --- | --- | --- | --- |
| FN | Chardonnay | Carneros | 4 | 14 |
| FN | Chardonnay | Carneros | 12 | 5 |
| FN | Chardonnay | Coombsville | 1 | 7 |
| FN | Chardonnay | Coombsville | 2 | 4 |
| FN | Chardonnay | Coombsville | 5 | 5 |
| FN | Chardonnay | Coombsville | 6 | 6 |
| FN | Chardonnay | Coombsville | 7 | 2 |
| FN | Chardonnay | Coombsville | 8 | 16 |
| FN | Chardonnay | Oakville | 10 | 6 |
| FN | Cabernet Sauvignon | Oakville | 10 | 37 |
| FN | Cabernet Sauvignon | Oakville | 18 | 4 |
| NN | Chardonnay | Carneros | 12 | 10 |
| NN | Chardonnay | Russian_River | 9 | 6 |
| NN | Chardonnay | Russian_River | 11 | 6 |
| NN | Cabernet Sauvignon | Oakville | 10 | 2 |
| NN | Cabernet Sauvignon | Oakville | 13 | 8 |
| NN | Cabernet Sauvignon | Oakville | 18 | 9 |
| NN | Cabernet Sauvignon | Oakville | 22 | 6 |
| NN | Cabernet Sauvignon | Oakville | 23 | 8 |
| NN | Cabernet Sauvignon | Rutherford | 14 | 11 |
| NN | Cabernet Sauvignon | Rutherford | 19 | 4 |
| NN | Cabernet Sauvignon | St. Helena | 16 | 3 |
| NN | Cabernet Sauvignon | St. Helena | 17 | 6 |
| NN | Cabernet Sauvignon | Yountville | 21 | 10 |

* FN = Far Niente; NN = Nickel & Nickel. Vineyard indicates the vineyard code where the grapes were grown. Count indicates the number of unique must samples collected from that vineyard.
