# Supplementary material for: Associations among Wine Grape Microbiome, Metabolome, and Fermentation Behavior Suggest Microbial Contribution to Regional Wine Characteristics
Source: mBio. 2016 Jun 14;7(3):e00631-16. doi: 10.1128/mBio.00631-16 (PMC4959672; doi:10.1128/mBio.00631-16)
Supplement: TABLE S2 — Permutational MANOVA comparisons of microbial diversity in musts and wines. [file mbo003162841st2.docx]

**Table S2. Permutational MANOVA Comparisons of Weighted Unifrac (WU) Distance and Bray-Curtis Dissimilarity (BC) Microbial Diversity in Musts and Wines**

|  |  |  | **Bacteria WU** | | **Fungi BC** | |
| --- | --- | --- | --- | --- | --- | --- |
| **Variety** | **Stage** | **Test** | ***R^2^*** | ***P*** | ***R^2^*** | ***P*** |
| Cabernet | Must | Region | 0.154 | < 0.001 | 0.105 | 0.002 |
|  |  | Vineyard | 0.353 | < 0.001 | 0.320 | < 0.001 |
|  | Wine (MLF) | Region | 0.029 | 0.749 | 0.129 | 0.160 |
|  |  | Vineyard | 0.156 | 0.342 | 0.523 | < 0.001 |
|  | All | Stage | 0.529 | < 0.001 | 0.377 | < 0.001 |
| Chardonnay | Must | Region | 0.262 | < 0.001 | 0.233 | < 0.001 |
|  |  | Vineyard | 0.599 | < 0.001 | 0.408 | < 0.001 |
|  | Wine (End) | Region | 0.162 | < 0.001 | 0.076 | 0.733 |
|  |  | Vineyard | 0.463 | < 0.001 | 0.245 | 0.766 |
|  | All | Stage | 0.170 | < 0.001 | 0.179 | < 0.001 |
| NN Chardonnay | All | Vineyard | 0.429 | < 0.001 | 0.164 | 0.020 |

NN = Nickel & Nickel winery; MLF = post-malolactic fermentation stage.
