# Supplementary material for: Associations among Wine Grape Microbiome, Metabolome, and Fermentation Behavior Suggest Microbial Contribution to Regional Wine Characteristics
Source: mBio. 2016 Jun 14;7(3):e00631-16. doi: 10.1128/mBio.00631-16 (PMC4959672; doi:10.1128/mBio.00631-16)
Supplement: TABLE S3 — Random forest models predict vineyard origin of grape musts. [file mbo003162841st3.docx]

**Table S3. Random Forests Models Predict Vineyard Origin of Grape Musts**

|  | Error Rate^a^ | Random Rate^b^ | ER/Random^c^ |
| --- | --- | --- | --- |
| Cabernet Must | 17.05 | 75.00 | 4.40 |
| Cabernet Wine | 19.35 | 85.71 | 4.43 |
| Chardonnay Juice | 20.45 | 85.71 | 4.19 |
| Chardonnay Wine | 17.65 | 66.00 | 3.74 |

^a^Error Rate = percentage of misclassification of out-of-bag must samples to the wrong vineyard.

^b^Random Rate = percentage of misclassification expected due to random error

^c^ER/Random = Error Rate / Random Rate.
