# Supplementary material for: Associations among Wine Grape Microbiome, Metabolome, and Fermentation Behavior Suggest Microbial Contribution to Regional Wine Characteristics
Source: mBio. 2016 Jun 14;7(3):e00631-16. doi: 10.1128/mBio.00631-16 (PMC4959672; doi:10.1128/mBio.00631-16)
Supplement: TABLE S4 — Regionally differential Cabernet Sauvignon wine mass features and putative metabolites. [file mbo003162841st4.docx]

**Table S4. Regionally Differential Cabernet Sauvignon Wine Mass Features and Putative Metabolites**

| **NM*** | **Mass** | **RT** | **Formula** | **Match** | **STD** | **MS/MS** | **Database Hits** |
| --- | --- | --- | --- | --- | --- | --- | --- |
| 130 A | 130.0263 | 2.0141 | C5H6O4 |  |  |  | C5 diacid or keto acid; acetyl pyruvate mass match, but no spectrum in database |
| 130 B | 130.0617 | 3.1302 | C6H10O3 |  |  |  | C6 ketoacid, no spectrum match in database |
| 136 | 136.0516 | 2.3515 | C8H8O2 |  |  |  | No spectra in database; possible aldehyde |
| 144 | 144.0409 | 1.0070 | C6H8O4 |  |  |  | C6 diacid, no MS/MS matches |
| 148 | 148.0711 | 0.7311 | C6H12O4 |  |  |  | C6 hydroxy acid, possibly mevalonic acid. |
| 152 A | 152.0095 | 4.1587 | C5H4N4S? |  |  |  | Mercaptopurine? MS match, incomplete MS/MS match |
| 152 B | 152.0096 | 3.3671 | C5H4N4S? |  |  |  | Mercaptopurine? MS match, incomplete MS/MS match |
| 154 | 154.0259 | 2.3046 | C7H6O4 |  |  |  | MS match for a dihydroxy benzoic acid, but no spectral matches in database |
| 170 | 170.0200 | 3.3671 | C7H6O5 |  |  |  | No spectra in database |
| 180 | 180.0414 | 2.3515 | C9H8O4 | X | X | X | Caffeic acid |
| 198 | 198.0513 | 3.2016 | C9H10O5 | X | X | X | Syringic acid |
| 205 | 205.0725 | 3.2640 | C11H11NO3 | X |  | X | Indolelactic acid |
| 208 | 208.0723 | 5.1325 | C11H12O4 | X |  | X | Ethyl-caffeate |
| 290 | 290.0774 | 2.9473 | C15H14O6 | X | X | X | Epicatechin |
| 294 A | 294.1302 | 2.6684 | C12H22O8 | X |  | X | Ethyl-glucopyranosyl butanoate |
| 294 B | 294.1821 | 7.2817 | C17H26O4 |  |  |  | No MS/MS matches |

*NM = nominal mass; Mass = accurate mass; RT = liquid chromatography run time (min); Formula = molecular formula for exact or putative matches; Match = “X” marks exact matches; STD = “X” marks accurate RT and mass match to authentic standard; MS/MS = “X” marks accurate match to MS/MS spectrum in Metlin database; Database Hits = confirmed compound identity for compounds marked in the Match column, or putative identities for unknown compounds, when available.
