# Supplementary material for: Associations among Wine Grape Microbiome, Metabolome, and Fermentation Behavior Suggest Microbial Contribution to Regional Wine Characteristics
Source: mBio. 2016 Jun 14;7(3):e00631-16. doi: 10.1128/mBio.00631-16 (PMC4959672; doi:10.1128/mBio.00631-16)
Supplement: TABLE S5 — Regionally differential chardonnay wine mass features and putative metabolites. [file mbo003162841st5.docx]

**Table S5. Regionally Differential Chardonnay Wine Mass Features and Putative Metabolites**

| **NM*** | **Mass** | **RT** | **Formula** | **Match** | **STD** | **MS/MS** | **Database Hits** |
| --- | --- | --- | --- | --- | --- | --- | --- |
| 102 | 102.0292 | 0.9364 | C4H6O3 |  |  |  | C4 keto acid, e.g. acetoacetic, methyl pyruvate, succinic semi-aldehyde |
| 110 | 110.0394 | 1.1499 | C6H6O2 |  |  |  | No MS/MS matches; accurate mass match for pyrocatechol or 5-methyl furfural |
| 114 | 114.0702 | 2.2747 | C6H10O2 |  |  |  | No spectra in database. C6H10O2 acid, ester, lactone--34 mass matches |
| 116 | 116.0510 | 2.2747 | C6H12O2? |  |  |  | No database matches |
| 120 | 120.0577 | 3.3848 | C8H8O |  |  |  | No MS/MS in database; possibly acetophenone, phenylacetaldehyde, or 3-methyl benzaldehyde |
| 126 | 126.0346 | 0.6034 | C6H6O3 | X |  | X | pyrogallol (1,2,3 trihydroxybenzene) |
| 130 | 130.0632 | 3.5069 | C6H10O3 |  |  |  | C6 ketoacid or lactone, no spectrum matches |
| 131 | 131.0968 | 0.5439 | C6H13NO2 |  |  |  | Probably L-leucine. Possible spectral match |
| 136 A | 136.0498 | 0.9307 | C8H8O2 |  |  |  | Probably C8H8O2, possibly methylbenzoate, phenyl acetate, p-Anisealdehyde |
| 136 B | 136.0524 | 2.7086 | C8H8O2 |  |  |  | No spectra in database; possible aldehyde |
| 144 A | 144.0435 | 1.2792 | C6H8O4 |  |  |  | C6 diacid, no MS/MS matches; dimethyl maleate, methylglutaconic acid are examples |
| 144 B | 144.1169 | 7.3606 | C8H16O2 | X | X |  | Octanoic acid |
| 150 A | 150.0139 | 0.9466 | C4H6O6 | X | X | X | Tartaric acid |
| 150 B | 150.0141 | 1.7050 | C4H6O6 |  |  |  | No good matches; possibly meso-tartaric acid |
| 154 | 154.0630 | 2.1130 | C7H6O4 |  |  |  | MS match for a dihydroxy benzoic acid, but no spectral matches in database |
| 164 A | 164.0442 | 1.7046 | C9H8O3 |  |  |  | Coumaric acid |
| 164 B | 164.0446 | 1.4888 | C9H8O3 |  |  |  | Coumaric acid |
| 170 | 170.0245 | 0.6096 | C7H6O5 | X | X | X | Gallic acid |
| 172 | 172.1518 | 8.6283 | C10H20O2 | X | X |  | Decanoic acid |
| 180 A | 180.0395 | 0.9286 | C9H8O4 |  |  |  | Caffeic acid, but RT not right |
| 180 B | 180.0426 | 2.7085 | C9H8O4 | X | X | X | Caffeic acid |
| 182 | 182.0579 | 0.9301 | C9H10O4 |  |  |  | Mass match for syringaldehyde, but wrong RT; homovanillic or veratric acid are mass matches |
| 198 | 198.0528 | 3.5425 | C9H10O5 | X |  | X | Ethyl gallate |
| 204 | 204.1179 | 5.5837 | C13H16O2 |  |  |  | Possibly mercaptol hexyl butyrates |
| 254 | 254.1582 | 8.6283 | C14H22O4 |  |  |  | No database matches |
| 272 | 272.1797 | 5.7748 | C16H16O4 |  |  |  | No database matches |
| 290 | 290.0804 | 2.4872 | C15H14O6 | X | X | X | Catechin |

*NM = nominal mass; Mass = accurate mass; RT = liquid chromatography run time (min); Formula = molecular formula for exact or putative matches; Match = “X” marks exact matches; STD = “X” marks accurate RT and mass match to authentic standard; MS/MS = “X” marks accurate match to MS/MS spectrum in Metlin database; Database Hits = confirmed compound identity for compounds marked in the Match column, or putative identities for unknown compounds, when available.
