# Supplementary material for: Associations among Wine Grape Microbiome, Metabolome, and Fermentation Behavior Suggest Microbial Contribution to Regional Wine Characteristics
Source: mBio. 2016 Jun 14;7(3):e00631-16. doi: 10.1128/mBio.00631-16 (PMC4959672; doi:10.1128/mBio.00631-16)
Supplement: TABLE S6 — Chardonnay metabolite random forest model summaries. [file mbo003162841st6.docx]

**Table S6. Chardonnay Metabolite Random Forest Model Summaries**

| ID^a^ | *R*^2^ | Top Features, descending order of importance |
| --- | --- | --- |
| 102.0292 | 0.60 | *Methylobacterium, Stenotrophomonas, Microbacteriaceae:Other, Curtobacterium, Sphingomonas, Bacillaceae, Bacillaceae:Other, Enterococcus* |
| 110.0394 | 0.55 | *Botryotinia fuckeliana, Pseudomonas, Bensingtonia, Sclerotinia sclerotiorum* |
| 114.0702 | 0.80 | *Pichia guilliermondii, Methylobacterium, Bifidobacterium, Acremonium, Penicillium, Comamonadaceae, Leuconostoc, Lactobacillus, Luteibacter, Gluconobacter, Enterococcus, Aureobasidium pullulans, Stenotrophomonas, Leuconostocaceae, Lactococcus, Hanseniaspora uvarum* |
| 116.0510 | 0.65 | *Methylobacterium, Acremonium, Stenotrophomonas, Acinetobacter, Lactococcus, Pseudomonadaceae, Enterobacteriaceae, Cladosporium, Erwinia, Leuconostoc, Oxalobacteraceae, Comamonadaceae, Luteibacter, Citrobacter, Achromobacter* |
| 120.0577 | 0.86 | *Leuconostoc, Alcaligenaceae, Leuconostocaceae, Xanthomonadaceae:Other, Lactobacillus, Lactococcus, Acremonium, Kaistobacter, Bifidobacterium, Acetobacter, Hanseniaspora uvarum, Gluconobacter, Penicillium, Achromobacter, Pichia guilliermondii, Planococcus, Bacillales, Xanthomonadaceae, Pseudomonas, Enterobacteriaceae:Other, Erwinia, Anoxybacillus, Acinetobacter, Enhydrobacter, Sphingobium, Halomonas, Wickerhamomyces anomalus, Enterobacteriaceae, Curtobacterium, Arcobacter, Lachnospiraceae, Sphingomonas* |
| 126.0346 | 0.66 | *Planococcus, Enhydrobacter, Bacillales, Brevundimonas, Alcaligenaceae, Lactobacillaceae, Acetobacter, Exiguobacterium* |
| 130.0632 | 0.78 | *Citrobacter, Acetobacter, Gluconobacter, Leuconostoc, Leuconostocaceae, Lactobacillus, Bacillales, Bifidobacterium, Planococcus, Hanseniaspora uvarum, Acremonium, Alcaligenaceae, Lactococcus, Kaistobacter, Comamonas* |
| 131.0968 | 0.72 | *Sporobolomyces, Planococcus, Enterobacteriaceae:Other, Bacillaceae, Citrobacter, Enterobacteriaceae, Pseudomonadaceae, Erwinia, Leuconostocaceae, Ochrobactrum, Acinetobacter, Exiguobacterium, Pseudomonadaceae:Other, Botryotinia fuckeliana, Xanthomonadaceae:Other* |
| 136.0498 | 0.83 | *Bifidobacterium, Lactobacillus, Leuconostocaceae, Ochrobactrum, Comamonas, Achromobacter, Bacillales, Bacillus* |
| 136.0524 | 0.21 | *Kaistobacter, Comamonas, Planococcaceae, Cryptococcus* |
| 144.0435 | 0.37 | *Pseudomonadaceae:Other, Pasteurellales, Pseudomonadaceae, Comamonas, Microbacteriaceae:Other, Oxalobacteraceae, Microbacteriaceae, Sphingomonas, Stenotrophomonas, Methylobacterium, Citrobacter, Pedobacter, Sphingobacteriaceae, Bacillales, Lactobacillaceae* |
| 144.1169 | 0.53 | *Stenotrophomonas, Enterococcus, Acetobacteraceae, Pseudomonas, Leuconostoc, Leuconostocaceae, Pichia guilliermondii, Lactobacillus, Acetobacter, Gluconobacter, Acremonium, Wickerhamomyces anomalus, Alcaligenaceae, Comamonas, Bifidobacterium, Penicillium* |
| 150.0139 | 0.80 | *Exiguobacterium, Comamonas, Bifidobacterium, Geobacillus, Achromobacter, Bacillales, Leuconostoc, Leuconostocaceae, Kaistobacter, Alcaligenaceae, Planococcus, Lactobacillus, Gluconobacter, Acetobacter, Xanthomonadaceae:Other, Acremonium, Lactococcus, Penicillium, Pichia guilliermondii, Hanseniaspora uvarum, CandidatusPortiera, Burkholderiales, Bacillus, Microbacteriaceae:Other, Sphingomonas, Bacillaceae:Other, Curtobacterium, Bacillaceae, Ochrobactrum, Planococcaceae* |
| 150.0141 | 0.31 | *Methylobacterium, Stenotrophomonas, Microbacteriaceae:Other, Sphingomonas, Curtobacterium, Enterococcus, Botryotinia fuckeliana, Sclerotinia sclerotiorum, Bacillaceae, Bacillaceae:Other, Comamonas, Bifidobacterium, Pedobacter, Pseudomonadaceae:Other, Kaistobacter* |
| 154.0630 | 0.81 | *Pseudomonas, Bensingtonia, Leuconostocaceae, Enterobacteriaceae* |
| 164.0442 | 0.52 | *Methylobacterium, Microbacteriaceae:Other, Sphingomonas, Curtobacterium* |
| 164.0446 | 0.97 | *Lactococcus, Leuconostoc, Lactobacillus, Acremonium, Alcaligenaceae, Penicillium, Leuconostocaceae, Acetobacter, Gluconobacter* |
| 170.0245 | 0.66 | *Planococcus, Bacillales, Lactobacillaceae, Acetobacter, Alcaligenaceae, Brevundimonas, Enhydrobacter, Exiguobacterium* |
| 172.1518 | 0.24 | *Sporobolomyces, Cryptococcus, Xanthomonadaceae:Other, Davidiella, Erwinia, Enterobacteriaceae, Acinetobacter, Alternaria* |
| 180.0395 | 0.83 | *Exiguobacterium, Bifidobacterium, Comamonas, Bacillales, Leuconostoc, Acetobacter, Lactobacillus, Leuconostocaceae* |
| 180.0426 | 0.27 | *Kaistobacter, Cryptococcus, Comamonas, Planococcaceae, Ochrobactrum, Bacillaceae, Citrobacter, Alcaligenaceae* |
| 182.0579 | 0.60 | *Achromobacter, Methylobacterium, Microbacteriaceae:Other, Leuconostoc, Pseudomonas, Bensingtonia, Bifidobacterium, Lactobacillus* |
| 198.0528 | 0.62 | *Enhydrobacter, Lactobacillaceae, Brevundimonas, Alcaligenaceae* |
| 204.1179 | 0.85 | *Microbacteriaceae:Other, Comamonas, Sphingomonas, Bacillus, Leuconostocaceae, Bacillaceae, Ochrobactrum, Curtobacterium, Leuconostoc, Achromobacter, Pediococcus, Oxalobacteraceae, Davidiella, Bifidobacterium, Sporobolomyces* |
| 254.1582 | 0.26 | *Sporobolomyces* |
| 272.1797 | 0.89 | *Comamonas, Bifidobacterium, Bacillales, Geobacillus* |
| 290.0804 | 0.73 | *Curtobacterium, Sphingomonas, Microbacteriaceae:Other, Stenotrophomonas, Oxalobacteraceae, Achromobacter, Alcaligenaceae, Hanseniaspora uvarum, Lactobacillus, Leuconostoc, Pichia guilliermondii, Bifidobacterium, Bacillales, Leuconostocaceae, Comamonas, Gluconobacter, Lactococcus, Xanthomonadaceae:Other, Planococcus, Acetobacter, Acremonium, Kaistobacter, Penicillium, Methylobacterium, Microbacteriaceae, Hymenobacter, Pediococcus, Lachancea, Geobacillus, Planococcaceae,* |

^a^ID = accurate mass of QTOF metabolites; *R*^2^ = pseudo *R*^2^ model accuracy; Nvar = number of optimal variables used in final model.
