# Supplementary material for: Associations among Wine Grape Microbiome, Metabolome, and Fermentation Behavior Suggest Microbial Contribution to Regional Wine Characteristics
Source: mBio. 2016 Jun 14;7(3):e00631-16. doi: 10.1128/mBio.00631-16 (PMC4959672; doi:10.1128/mBio.00631-16)
Supplement: TABLE S7 — Cabernet Sauvignon metabolite random forest model summaries. [file mbo003162841st7.docx]

**Table S7. Cabernet Sauvignon Metabolite Random Forest Model Summaries**

| ID^a^ | *R*^2^ | Top Features, descending order of importance |
| --- | --- | --- |
| 130.0263 | 0.46 | *Xanthomonadaceae, Erwinia, Hymenobacter, Stemphylium, Arthrobacter, Leptosphaeria, Planococcaceae, Pseudomonas, Novosphingobium, Saccharomyces cerevisiae, Cryptococcus, Lactobacillus* |
| 130.0617 | 0.60 | *Cladosporium, Bacillaceae, Saccharomyces cerevisiae, Bacillus, Aureobasidium pullulans, Novosphingobium, Comamonas, Xanthomonadaceae, Legionellales, Rhodotorula nothofagi, Curtobacterium, Wickerhamomyces anomalus, Hymenobacter, Pseudomonas, Exiguobacterium, Bacillaceae:Other* |
| 136.0516 | 0.42 | *Enterobacteriaceae:Other, Phlebia, Leuconostocaceae, Rhodotorula* |
| 144.0409 | 0.40 | *Stemphylium, Arthrobacter, Hymenobacter, Leptosphaeria, Xanthomonadaceae, Novosphingobium, Aureobasidium pullulans, Enterobacteriaceae:Other, Leuconostocaceae, Acinetobacter, Lachancea, Phlebia, Pseudomonas, Cryptococcus, Comamonas, Sphingobacteriaceae* |
| 148.0711 | 0.47 | *Acinetobacter, Bacillaceae:Other, Gluconobacter, Acetobacteraceae, Pasteurellales, Bacillus, Botryotinia fuckeliana, Bacillaceae, Aureobasidium pullulans, Pseudomonas, Xanthomonadaceae, Sporobolomyces, Erwinia, Saccharomyces cerevisiae, Cladosporium, Udeniomyces* |
| 152.0095 | 0.22 | *Aureobasidium pullulans, Pichia guilliermondii, Legionellales, Bensingtonia* |
| 152.0096 | 0.51 | *Aureobasidium pullulans, Methylobacterium, Candida zemplinina, Lactobacillaceae:Other, Bacillales, Comamonas, Legionellales, CandidatusPortiera, Phlebia, Acetobacter, Stemphylium, Cladosporium, Bensingtonia, Rickettsiella, Pichia guilliermondii, Mycosphaerella* |
| 154.0259 | 0.16 | *Bacillus, Penicillium, Alternaria alternata, Blautia* |
| 170.02 | 0.50 | *Aureobasidium pullulans, Methylobacterium, Legionellales, Lactobacillaceae:Other, Candida zemplinina* |
| 180.0414 | 0.41 | *Enterobacteriaceae:Other, Phlebia, Lactobacillus, Aureobasidium pullulans, Leuconostocaceae, Comamonadaceae:Other, Acinetobacter, Comamonas, Methylobacterium, Pseudomonadaceae, CandidatusPortiera, Erwinia, Incertae sedis, Peniophora, Candida, Enterococcus* |
| 198.0513 | -0.04 | *Acinetobacter, Gluconobacter, Leuconostocaceae, Rhodotorula nothofagi, Peniophora, Xanthomonadaceae, Bacillaceae, Pasteurellales, Wickerhamomyces anomalus, Pichia guilliermondii, Botryotinia fuckeliana, Xanthomonadaceae:Other, Lactobacillus, Enterobacteriaceae:Other, Bifidobacterium, Pseudomonadaceae:Other, Acetobacteraceae, Ochrobactrum, Achromobacter, Bacillus, Udeniomyces, Phlebia, Sporobolomyces, Hanseniaspora uvarum, Polyporales, Cryptococcus, Oxalobacteraceae, Acetobacter, Saccharomyces cerevisiae, Lactococcus, Aureobasidium pullulans* |
| 205.0725 | 0.11 | *Enterobacteriaceae, Enterobacteriaceae:Other, Hanseniaspora uvarum, Saccharomyces cerevisiae, Sclerotiniaceae, Erwinia, Methylobacterium, Pichia guilliermondii* |
| 208.0723 | 0.27 | *Brevundimonas, Arthrobacter, Alicyclobacillus, Oxalobacteraceae, Blautia, Sclerotinia sclerotiorum, Sphingomonas, Saccharomyces cerevisiae, Bacillaceae, Botryotinia fuckeliana, Candida, Sporobolomyces, Alternaria, Hanseniaspora uvarum, Erwinia, Fructobacillus* |
| 290.0774 | 0.51 | *Bacillaceae, Bacillus, Alternaria alternata, Blautia, Filobasidiaceae, Pseudomonas, Saccharomyces cerevisiae, Alternaria, Candida, Sphingobacteriaceae, Wickerhamomyces anomalus, Oxalobacteraceae, Sphingomonas, Planifilum, Incertae sedis, Penicillium, Acetobacteraceae, Root, Bacillaceae:Other, Sclerotinia sclerotiorum, Pasteurellales, Acinetobacter, Acremonium, Botryotinia fuckeliana, Peniophora, Cladosporium, Hanseniaspora uvarum, Polyporales, Ulocladium, Seimatosporium, Xanthomonadaceae* |
| 294.1302 | 0.05 | *Sphingomonas, Bacillales, Lactobacillaceae:Other, Exiguobacterium, Pasteurellales, Botryotinia fuckeliana, Acinetobacter, Aureobasidium pullulans* |
| 294.1821 | 0.46 | *Wickerhamomyces anomalus, Leuconostocaceae, Cryptococcus macerans, Lachancea, Saccharomyces cerevisiae, Leptosphaeria, Aureobasidium pullulans, Alternaria alternata,* |

^a^ID = accurate mass of QTOF metabolites; *R*^2^ = pseudo *R*^2^ model accuracy; Nvar = number of optimal variables used in final model.
